# Recycled Polyurethane Glycolysate and Glycerolysate as Sustainable Plasticizers for Lignin-Filled NBR Composites

**DOI:** 10.3390/ma19061204

**Published:** 2026-03-19

**Authors:** Ján Kruželák, Michaela Džuganová, Katarína Tomanová, Roderik Plavec, Paulina Parcheta-Szwindowska, Marcin Włoch, Magdalena Bąk, Janusz Datta

**Affiliations:** 1 Institute of Natural and Synthetic Polymers, Faculty of Chemical and Food Technology, Slovak University of Technology in Bratislava, Radlinského 9, 81237 Bratislava, Slovakiaroderik.plavec@stuba.sk (R.P.); 2 Department of Polymers Technology, Faculty of Chemistry, Gdańsk University of Technology, G. Narutowicza Str. 11/12, 80233 Gdańsk, Polands185286@student.pg.edu.pl (M.B.)

**Keywords:** chemical recycling, polyurethane glycerolysate, polyurethane glycolysate, sustainable rubber composites, lignosulfonate, lignin

## Abstract

Glycolysate and glycerolysate—organic substances recovered from the chemical recycling of polyurethane waste—were investigated as sustainable plasticizers for acrylonitrile-butadiene rubber composites filled with 30 phr of calcium lignosulfonate or kraft lignin. The study evaluated the impact of these recycled plasticizers (added at 10 and 15 phr) on the curing process, morphology, rheology, mechanical and dynamic mechanical performances. Rheological analysis confirmed that both plasticizers significantly reduced the complex viscosity of the rubber compounds, with the effect being most pronounced at the 15 phr loading. While the incorporation of glycolysate and glycerolysate slightly extended the optimum cure time and decelerated the curing process, the cross-link density remained consistently within the range of 3.5–4 × 10^−4^ mol·cm^−3^. Morphological studies revealed that the plasticizers facilitated better dispersion of both lignin types and improved interfacial adhesion. However, the mechanical response differed significantly depending on the filler type. A consistent increase in elongation at break was observed only for composites filled with kraft lignin, where values rose from 341% for the reference up to 571% for the sample with 15 phr of glycolysate. In contrast, the application of plasticizers to calcium lignosulfonate-filled composites led to an initial decrease in both tensile strength and elongation at break. Notably, kraft lignin-filled composites exhibited superior overall mechanical performance, with glycolysate effectively maintaining tensile strength levels comparable to the reference. While both recovered substances performed effectively as processing aids, they had a negligible effect on the glass transition temperature. The results demonstrated that these recovered polyurethane derivatives are highly effective, sustainable alternatives to conventional plasticizers, showing a clear synergistic effect particularly with kraft lignin.

## 1. Introduction

Today, more than ever, the scientific community should recognize the necessity of conducting extensive research aimed at developing materials useful for industry, utilizing secondary raw materials (r-raw materials) derived from natural-based sources, used products or post-production waste. The concept of product circularity should become increasingly evident in the mass production of goods made from various materials, including natural-based products or waste plastics, which allows for reducing the consumption of environmentally harmful fossil resources. Moreover, global warming, climate change, and adverse environmental impacts have redirected wide attention toward bioeconomic frameworks and sustainable technological innovations.

Lignocellulosic feedstocks constitute viable substitutes for petroleum-derived materials, given their abundant availability, renewable nature, and beneficial contribution to the mitigation of global greenhouse gas emissions. As the second most abundant biopolymeric substance globally, lignin possesses considerable potential for diverse applications. Annual technical lignin production ranges from approximately 50 to 70 million tons, yet merely 1–2% of this quantity is utilized for manufacturing value-added products. The remaining portion is either disposed of in landfills or combusted for energy generation or chemical recovery purposes [1,2]. Lignin demonstrates remarkable properties, such as exceptional mechanical and thermal stability, favorable physico-mechanical characteristics, as well as adhesive, antioxidant, anti-UV, and antimicrobial attributes [3,4,5,6,7,8]. Its eco-friendliness, capacity for biodegradation, extensive ecological adaptability, and enhancement characteristics position it as a superior substitute for the fabrication of innovative environmentally sustainable polymer composites [9,10,11,12,13,14]. Lignin exhibits an amorphous, three-dimensional configuration characterized by extensive aromatic branching. The fundamental structural components of lignin comprise phenylpropane units, specifically p-hydroxyphenyl (H), syringyl (S), and guaiacyl (G) moieties, which are interconnected through carbon-carbon (C–C) linkages and ether (C–O) bonds [15,16]. Various extraction methodologies and delignification procedures enable the isolation of lignin from various plants, including agricultural crops and woody biomass. The specific extraction technique employed determines the resulting chemical composition and physico-chemical properties of the recovered lignin fraction.

Biomass undergoes diverse physical, chemical, and biochemical methodologies for the isolation and extraction of lignin. These extraction methodologies are fundamentally categorized into sulfur-containing and sulfur-free approaches [17,18,19,20]. Sulfur-based methodologies demonstrate greater prevalence in industrial applications, with the resulting sulfur-bearing lignin derivatives encompassing Kraft lignins and lignosulfonates. The sulfite pulping method employs an acid-catalyzed mechanism operating within a pH range of 1–2 at temperatures of 140–170 °C, utilizing an aqueous sulfur dioxide solution in conjunction with acid compounds derived from sodium, magnesium, calcium, or ammonium salts [21]. Throughout this procedure, the breakdown and following sulfonation of ether linkages within the lignin macromolecular structure takes place, resulting in the formation of lignosulfonates characterized by comparatively elevated molecular weights ranging from 15,000 to 60,000 daltons and diminished concentrations of phenolic functional moieties. Lignosulfonates exhibit a higher concentration of sulfur-bearing functional groups, predominantly manifested as SO_3_^2−^ and HSO_3_^−^ moieties, which confer them anionic characteristics and aqueous solubility [18,22,23,24]. Conversely, relative to Kraft lignins, lignosulfonates demonstrate a reduced amount of phenolic hydroxyl and carboxyl functionalities. Kraft lignin is derived through a sulfate-based delignification procedure (Kraft pulping) wherein the ether linkages within lignin macromolecular structures undergo cleavage using sodium hydroxide and sodium sulfide, resulting in the formation of black liquor characterized by an extremely alkaline pH ranging from 13 to 14. Additional purification techniques yield kraft lignin characterized by a broad molecular weight distribution, with values falling within a lower range compared to lignosulfonates (200–20,000 Da). The resulting kraft lignin exhibits water-insoluble properties and demonstrates a comparatively elevated content of phenolic functional groups [25,26,27,28].

A significant concentration of carbon, along with mechanical stability and favorable rheological and viscoelastic characteristics, renders lignins and lignosulfonates appropriate candidates for use as fillers and additives in rubber formulations [29,30,31,32,33,34,35]. However, lignin and its derivatives possess numerous functional groups, including hydroxyl, methoxyl, carboxyl, carbonyl functional groups or sulfur-derived moieties, which facilitate the formation of robust intramolecular and intermolecular interactions among lignin macromolecules. Furthermore, the amorphous heterogeneous characteristics and highly branched architectural framework impede their compatibility and miscibility with most polymers. Poor compatibility and adhesion between the polymer and the filler on their interface often result in inferior physico-mechanical characteristics of the final composites. One of the ways to improve the dispersion of the biopolymers within the polymer matrices and improve the adhesion and homogeneity on the interfacial polymer-filler region is to use the compatibilizers or plasticizers. In general, plasticizers are typically employed to enhance the processability of polymer compounds by reducing melt viscosity and facilitating improved dispersion and distribution of fillers and functional additives. Consequently, these agents contribute to the formation of homogeneous polymer formulations. Furthermore, plasticizers enhance the molecular mobility of polymer chains and lower the glass transition temperature, thereby improving the elastic characteristics of the resultant materials and increasing their ultimate elongation. However, the incorporation of plasticizers frequently results in a reduction in the composites’ hardness and ultimate tensile strength properties. The application of suitable plasticizers also has a significant impact on the plasticization of lignin, mainly by weakening strong intramolecular and intermolecular physical interactions between the chain segments, thereby improving flexibility, chain mobility and melt-flow properties. This technique is crucial for overcoming lignin’s high glass transition temperature and rigid nature, enabling its use in thermoplastic applications and polymer composite materials [36,37]. The results of our previous works have revealed that the addition of suitable low molecular weight plasticizers, such as glycerol, 1,4-butanediol, ethylene glycol or sorbitol, enhanced the dispersion of lignin and improved the interfacial compatibility and adhesive properties between the biopolymer and rubber matrices, thereby facilitating improvements in processability and mechanical performance of the composite materials [38].

The literature contains only a few examples of using polyurethane recycling products (glycolysates/glycerolysates) as modifiers for rubber compounds and composites, e.g., in carbon black-filled natural rubber mixtures, where glycolysates influenced vulcanization kinetics and mechanical properties, and as glycerolysates modifying the properties of natural rubber compounds and vulcanizates [39,40]. Moreover, studies addressing the combined effect of both lignin and polyurethane glycolysates on vulcanizate properties are still scarce, indicating that the integration of these two resources has been rarely explored.

Nevertheless, such an approach aligns with the current global emphasis on developing “green” composites and multifunctional materials derived from natural biopolymers. The versatility of biomass-derived macromolecules is increasingly demonstrated across various environmental technologies, including the development of high-performance sustainable hydrogels for pollution management [41,42]. These advancements underline the growing importance of bio-based components, such as chitosan or lignin, in creating sustainable solutions that support the principles of a circular economy and environmental protection.

The reuse of recovered polymer components in the production process—such as oligomers or other products commonly referred to as recyclates (e.g., glycolysates or glycerolysates of polyurethane)—offers numerous benefits. Among these are waste reduction, lowering the carbon footprint of new products by at least 50%, as well as potential cost savings in production and increased environmental friendliness [43,44,45,46]. Many research centers worldwide are working on such solutions, which may be implemented in industrial practice in the future (examples include the activities of companies such as Covestro, BASF, Repoly, and RenCom).

The so-called “wet” chemical recycling belongs to more complex and technologically demanding processes, primarily due to the necessity of using components that initiate depolymerization and the complexity of the procedure itself. This applies, among others, to the chemical recycling (known as feedstock recycling, tertiary recycling) of polyurethane waste, which—through the action of glycol, triols, amines, water, or acids at elevated temperatures—leads to the breakdown of the polymer into smaller molecules. After proper stabilization or purification, these can be reintroduced into technological formulations, serving as valuable recovered reagents in chemical mixtures.

Covestro (France) subjects flexible furniture foams to glycolysis, obtaining high-purity polyol that is returned to production. The German company Repoly also uses glycolysis to produce polyols for new polyurethane foams. The Spanish company Repsoil processes polyurethane waste through acydolysis (5000 tons/year) and, since 2023, has produced a product called ReciclexR.

In general, the chemical depolymerization/degradation of polyurethane involves a transesterification reaction between the ester group attached to the carbonyl carbon of the urethane and the functional group of the splitting agent. During this reaction, chemical bonds between the carbon atom in the main chain and a heteroatom are broken, resulting in the formation of intermediates. The degradation process involves breaking urethane and urea bonds, as well as allophanate and biuret groups. The resulting intermediates can include repolyols—oligomeric compounds more or less similar to the original polyol—as well as by-products such as carbamates, low molecular weight diamines, and unreacted splitting agents. The reaction intermediates may terminate with hydroxyl and/or amine groups [47,48,49,50].

Based on the current state of knowledge, this study was focused on the application and evaluation of glycolysate and glycerolysate—recovered from the chemical recycling of polyurethane waste—as sustainable plasticizers in acrylonitrile-butadiene rubber composites filled with lignin and lignosulfonates. While these intermediates are traditionally reintegrated into the synthesis of new polyurethanes, their potential as functional additives in rubber formulations remains largely unexplored. The primary objective was to investigate the influence of these recycled plasticizers on the vulcanization kinetics, rheological behavior, morphology and properties of the resulting vulcanizates. By integrating biomass-derived fillers with chemically recycled modifiers, this research aims to provide a comprehensive understanding of the synergetic effects between the components, contributing to the development of more sustainable and high-performance “green” rubber composites.

## 2. Materials and Experiments

### 2.1. Materials

Acrylonitrile-butadiene rubber (NBR; SKN 3345, Sibur International, Moscow, Russia) with 31–35% acrylonitrile content was used as the matrix. The cross-linking of rubber compounds was carried out by the application of a sulfur vulcanization system consisting of 3 phr sulfur (Siarkopol, Tarnobrzeg, Poland), 1.5 phr N-cyclohexyl-2-benzthiazole sulfenamide (CBS) acting as an accelerator (Duslo, Šaľa, Slovakia), and 3 phr zinc oxide (ZnO) with 2 phr stearic acid (Slovlak, Košeca, Slovakia) serving as activators.

Two types of lignin, differing in the pulping method, were used as fillers: calcium lignosulfonate, designated as CaL (Borrement CA120, Borregaard Deutschland GmbH, Karlsruhe, Germany), and kraft lignin, specified as kL (BioPiva 100, UPM Biochemicals, Helsinki, Finland). The key physicochemical characteristics of the kL and CaL fillers are detailed in Table 1. The fillers were used as received; their complex macromolecular structure and tendency to aggregate limited the applicability of standard particle size distribution analysis.

Additionally, two types of plasticizers obtained via chemical recycling of polyurethane waste were used: a glycolysate referred to as GLE (GLE 1101), prepared from polyurethane foam (PUF), and a glycerolysate referred to as GCR (GCR 8101), prepared from cast polyurethane (CPU). The preparation and characterization of both plasticizers are described in the following sections.

### 2.2. Preparation of Plasticizers

The novel plasticizers investigated in this study were produced via chemical recycling of polyurethane waste, prepared in collaboration with the Gdańsk University of Technology. These recovered products, categorized as glycolysates (GLE) and glycerolysates (GCR), were derived from flexible polyurethane foam waste consisting of aromatic diisocyanate (MDI) and polyols (primarily polyether), along with standard PUR foam additives.

The synthesis was conducted in a steel reactor. Initially, the depolymerizing agent (ethylene glycol or crude glycerol) and the catalyst (potassium acetate) were introduced and preheated to 160 °C or 180 °C, respectively. Once the target temperature was reached, the crumbled polyurethane foam was added in batches to ensure complete dissolution. Throughout the depolymerization, the stirring speed and foam feed rate were adjusted according to the dissolution rate, maintaining a weight ratio of polyurethane waste to the depolymerizing agent at a maximum of 10:1. Based on this methodology, two distinct plasticizers were synthesized:

GLE 1101: A glycolysate obtained from flexible polyurethane foam using ethylene glycol as the depolymerizing agent. The reaction was maintained at 200 °C for 1 h.

GCR 8101: A glycerolysate prepared from cast polyesterurethane using crude glycerol (a biodiesel production by-product, 80% purity) as the depolymerizing agent. The reaction was conducted at 220–240 °C for approximately 1 h.

The higher temperature range for GCR 8101 (220–240 °C) compared to GLE 1101 (200 °C) stems from the differences in the thermal and chemical properties of the depolymerization agents used. The temperature of 200 °C for GLE is optimal due to the lower molecular weight and boiling point of ethylene glycol, which is effective within a narrower and lower temperature range. In contrast, glycerol is characterized by a higher boiling point and a multifunctional hydroxyl structure, requiring a higher and broader temperature range for the efficient depolymerization of the polyurethane network. This range was established through experimental optimization to ensure sufficient breakdown of the polyurethane waste (especially for the more rigidly cross-linked CPU) while avoiding excessive side reactions. While temperature fluctuations within this range can theoretically affect the molecular weight distribution and hydroxyl number, the process was conducted under strictly controlled conditions, as confirmed by the consistent properties and high reproducibility of the resulting products.

The chemical recycling process intentionally results in a heterogeneous mixture of oligomeric compounds. To provide a quantitative compositional breakdown of the recyclates, Gel Permeation Chromatography (GPC) was performed for the GCR plasticizer. The molecular weight distribution (MWD) confirmed the presence of diverse oligomeric species (Table 2), which is characteristic of high-yield chemical recycling products and crucial for their plasticizing efficiency.

Upon completion of the reaction and subsequent cooling to room temperature, the resulting polymer mass separated spontaneously into two distinct phases. The separation process was carried out in a separatory funnel for 24 h to ensure equilibrium. The interface was visually well-defined due to significant differences in density and color between the layers; the upper phase appeared translucent and lighter, while the lower phase was darker and more viscous. This phase separation was found to be highly reproducible across multiple batches, as confirmed by consistent hydroxyl value and viscosity measurements of the collected fractions. The upper phase, which represented approximately 80–85% of the total reaction product weight, was collected for further chemical structure analysis and property testing. The lower phase (approx. 15–20%), containing unreacted agents and heavier residues, was removed. The final GLE and GCR products (upper phase) predominantly consist of polyol oligomers incorporating both soft and hard segments, as well as residual urethane groups. Regarding the purity and catalyst residues, the concentration of alkali metal ions (Na^+^ and K^+^) in the final products is strictly controlled by the synthesis protocol, which includes neutralization and phase separation steps. Based on established industrial standards for polyether polyols and chemical recycling protocols [51], the total alkali residue is maintained at trace levels (typically below 10 ppm). This level of technical purity is indirectly validated by the consistent vulcanization kinetics observed across all tested samples (Section 3.2.2); significant ionic contamination would lead to erratic scorch times and curing rates, which were not detected in this study. This ensures that the observed plasticization and intermolecular interactions are governed by the oligomeric structure and not by residual impurities.

### 2.3. Fabrication of Rubber Composites

Rubber compounds were prepared using a laboratory kneading machine (Brabender GmbH & Co. KG, Duisburg, Germany) at 90 °C for GLE-containing compounds and 110 °C for those with GCR plasticizer. For each set, a reference sample was prepared at the corresponding temperature. The mixing process lasted 10 min and was conducted in two stages. In the first stage, lignin with plasticizer and ZnO with stearic acid were added; in the second stage, sulfur and CBS were incorporated. After each stage, the compounds were sheeted using a two-roll mill. The formulations of the prepared rubber compounds are summarized in Table 3. The concentration of biopolymer fillers (CaL and kL) was kept constant at 30 phr, while the recycled plasticizers (GLE and GCR) were applied at 10 phr and 15 phr. These concentrations were selected based on previous studies, which identified 30 phr of biopolymer and 15 phr of plasticizer as an optimal balance between processability and mechanical performance [48,49,50]. The sulfur-based curing system was maintained at a constant ratio across all formulations (as detailed in Section 2.1) to ensure that observed changes in properties result solely from the filler–plasticizer interactions. The mixing temperatures (90 °C for GLE and 110 °C for the GCR series) were selected based on processing optimization.

The higher temperature of 110 °C for the GCR series was found to be more effective in reducing the plasticizer’s viscosity, leading to improved filler wetting and more uniform dispersion within the NBR matrix. Both recycled polyols are products of high-temperature synthesis (above 200 °C) and are thermally stable at the compounding and curing temperatures (170 °C), with no risk of evaporation or degradation.

For clarity, the samples are designated based on the type of filler and plasticizer, followed by the plasticizer content (e.g., kL/GLE_10_). Reference samples without plasticizers were prepared for both lignin types at both mixing temperatures to provide an accurate baseline for comparison. Curing was carried out at 170 °C under a pressure of 15 MPa using a hydraulic press (Fontijne, Vlaardingen, The Netherlands), following the optimum cure time for each compound.

### 2.4. Testing Methods

#### 2.4.1. Testing of GLE and GCR Samples

Density was measured according to PN-EN ISO 1675. The measurement was carried out using a pycnometer. Measurements were carried out for three samples, and the results were averaged. Hydroxyl number of the recovered polyol was determined by a standard titration method (PN-EN ISO 2554:2001). Measurements were carried out for three samples, and the results were averaged.

Viscosity analyses were performed by using the rotary rheometer R/S-CPS+ (Brookfield, Middleboro, MA, USA). Viscosity measurements were performed at the following temperatures: 45 °C and 50 °C. Each measurement consisted of three measurement blocks, each lasting 180 s. During a single block, 30 measurement points were recorded. In the first part of the measurement, the sample was sheared with an increasing shear rate from 0 to 300 s^−1^. Next, it was tested at a constant shear rate of 300 s^−1^. In the final stage, the measurement was performed while the shear rate decreased from 300 s^−1^ to 0. From the results obtained, the average viscosity values were determined.

The thermal stability and degradation behavior of the recycled plasticizers were investigated by thermogravimetric analysis (TGA). The TG curves were recorded using a METTLER TOLEDO TGA/DSC 1 instrument (Schwerzenbach, Zurich, Switzerland). The samples (approx. 10 mg) were placed in alumina crucibles and heated from 25 °C to 800 °C at a constant heating rate of 10 °C/min. The measurements were conducted under a nitrogen atmosphere with a flow rate of 50 mL/min to prevent oxidative degradation during the initial heating phase.

#### 2.4.2. Testing of Composites

The curing characteristics were investigated using an oscillatory rheometer (MDR 2000, Alpha Technologies, Akron, OH, USA) according to the STN 62 1416 at a temperature of 170 °C. The crosslink density was determined by measuring the equilibrium swelling of the composites in toluene after 30 h at a laboratory temperature. The cross-link density *ν_ch_* was calculated using the Flory–Rehner equation modified by Kraus [52], based on the equilibrium swelling state:

$$\nu_{ch}=-\frac{V_{r0}}{V_{s}}\frac{\ln\left(1-V_{r}\right)+V_{r}+\chi V_{r}^{2}}{\left(V_{r}^{\frac{1}{3}}\cdot V_{r0}^{\frac{2}{3}}-0.5V_{r}\right)}$$

*ν_ch_* is the cross-link density (mol·cm^−3^)
*V_r0_* is the volume fraction of rubber in the equilibrium swollen sample of vulcanizate in the absence of filler
*V_r_* is the volume fraction of rubber in the equilibrium swollen sample of filled vulcanizate
*V_S_* is the molar volume of the solvent (for toluene = 106.3 cm^3^·mol^−1^)
*χ* is the Flory–Huggins interaction parameter (for NBR-toluene *χ* = 0.4253)

The Flory–Huggins interaction parameter for the NBR-toluene (*χ* = 0.4253) is widely accepted as a representative constant for NBR grades with medium acrylonitrile content (approx. 33–34%) in toluene at room temperature. While it is recognized that χ can be influenced by the specific ACN content and the presence of additives, the use of this standard value provides a consistent and reliable basis for evaluating the relative changes and trends in the cross-link density across the different investigated filler and plasticizer systems. It should be noted that the Flory–Rehner equation assumes ideal swelling behavior, which can be partially limited in heterogeneous filled systems where filler particles restrict the swelling of the elastomer matrix due to polymer–filler interactions. To ensure the validity of the results and account for these limitations, the Kraus correction was applied. This model specifically addresses the restricted swelling in filled systems by considering the volume fraction of the filler and its influence on the overall swelling ratio. By combining the Flory–Rehner equation with the Kraus correction, it was possible to separate the influence of the filler–rubber interface from the chemical cross-link density of the rubber matrix. While any swelling model in a multi-component system remains an approximation, this methodology is a well-established standard in rubber science for evaluating relative changes in network structure across different additive systems.

The mechanical properties were measured using a Zwick Roell/Z2.5 appliance (Zwick GmbH & Co. KG, Ulm, Germany) according to the ISO 37 standard. The tests were carried out at a crosshead speed of 500 mm.min^−1^. Dumbbell-shaped specimens (Type 2, 6.4 mm wide and 80 mm long) were prepared from 2 mm thick cured rubber sheets using a specialized cutting knife. The results are presented as an average of five parallel measurements ± standard deviation.

The surface morphology and microstructure of composites were observed using a scanning electron microscope JEOL JSM-7500F (Jeol Ltd., Tokyo, Japan). The samples were initially frozen in liquid nitrogen, fractured, and then coated with a layer of gold on the fracture surface. The electron source used was a cold cathode ultra-high vacuum (UHV) field emission gun. The acceleration voltage ranged from 0.1 to 30 kV, resulting in a resolution of 1.0 nm at 15 kV and 1.4 nm at 1 kV. Scanning electron microscopy (SEM) images were captured by a CCD-Camera EDS (INCA X-ACT, Oxford Instruments, Abingdon, UK).

## 3. Results and Discussion

### 3.1. Characterization of GLE and GCR

The fundamental characteristics of the applied plasticizers, including density, viscosity, and hydroxyl value, which are essential for understanding their interaction with the fillers and the matrix, are provided in Table 4.

**Figure 1:**
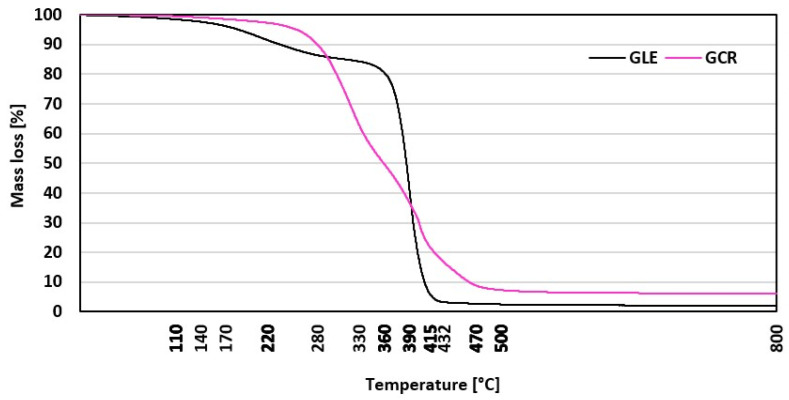
TG curves for plasticizers GLE and GCR.

Thermogravimetric analysis (TGA) of the two plasticizers, designated GLE (black curve) and GCR (pink curve), is presented in Figure 1. The plot shows the percentage of sample weight loss as a function of temperature. To quantitatively evaluate and compare the thermal stability of the samples, the characteristic temperatures at 5%, 10%, and 50% weight loss (T_5%_, T_10%_, and T_50%_) were determined and are summarized in Table 4. Both samples exhibit high thermal stability up to approximately 250 °C, maintaining nearly 100% of their initial mass. Beyond this temperature, significant thermal decomposition occurs. The GCR begins to lose weight earlier, with a T_5%_ of 255 °C, and degradation occurs over a broad temperature range from approximately 250 °C to 430 °C. In contrast, the GLE demonstrates greater thermal stability, with the onset of degradation (T_5%_) delayed until 338 °C. Its weight loss proceeds sharply over a narrower temperature range between 330 °C and 420 °C. Above approximately 470 °C, both samples reach a residual mass of about 5% or less (Table 5), indicating near-complete thermal decomposition. The observed differences in thermal degradation profiles suggest that both GLE and GCR have potential for use as plasticizers in rubber compounds, as their thermal stability significantly exceeds the temperatures required for compounding and vulcanization (up to 170 °C). In addition, due to the presence of hydroxyl and partially aromatic groups in their chemical structure, these glycolysates/glycerolysates can potentially act as natural antioxidant stabilizers in rubber compounds.

### 3.2. Characterization of Rubber Composites

#### 3.2.1. Predicted Mechanism of GLE and GCR in Rubber Compounds

The incorporation of GLE and GCR into the rubber matrix influences the system through several synergistic mechanisms. Primarily, as polyol fragments containing hydroxyl and residual urethane groups, these plasticizers form hydrogen bonds and dipole–dipole interactions with polar sites within the NBR matrix. This interaction enhances the compatibility between the polymer phase and the modifiers, effectively reducing compound viscosity and facilitating more uniform filler dispersion.

During the sulfur vulcanization process, these glycolysates and glycerolysates act as effective plasticizers by increasing the free volume and the mobility of the rubber chains. This molecular-level plasticization directly impacts the processing safety and curing kinetics, specifically affecting scorch time and curing rates. Although both plasticizers are polar, their distinct efficiencies are governed by their chemical architecture. GLE consists of glycol-based polyols with lower functionality, while GCR is composed of glycerol-based polyols characterized by a higher number of hydroxyl groups per molecule. This higher functionality of GCR increases the density of the hydrogen-bonding network, which restricts the mobility of the plasticizer molecules differently compared to GLE and modifies the filler–matrix interfacial interactions. These structural differences explain the variations in the plasticizing performance observed in the rheological and mechanical data.

Furthermore, the overall cohesion of the rubber matrix is enhanced through strong intermolecular forces. In the specific case of CaL-filled composites, ion-dipole interactions occur between the calcium ions in the filler and the hydroxyl groups of the plasticizers. These coordination effects, supported by the trace presence of residual catalytic ions (K^+^/Na^+^) at levels below 10 ppm, further promote filler–plasticizer compatibility and interfacial adhesion. The high purity of the recycled products ensures that these interactions are dominated by the oligomeric structure rather than catalyst residues. Consequently, the resulting vulcanizates demonstrate a balance of enhanced elasticity and robust mechanical properties, provided that the concentration of the oligomers is optimized to maintain effective stress transfer within the composite system.

#### 3.2.2. Curing Process and Cross-Link Density

**Figure 2:**
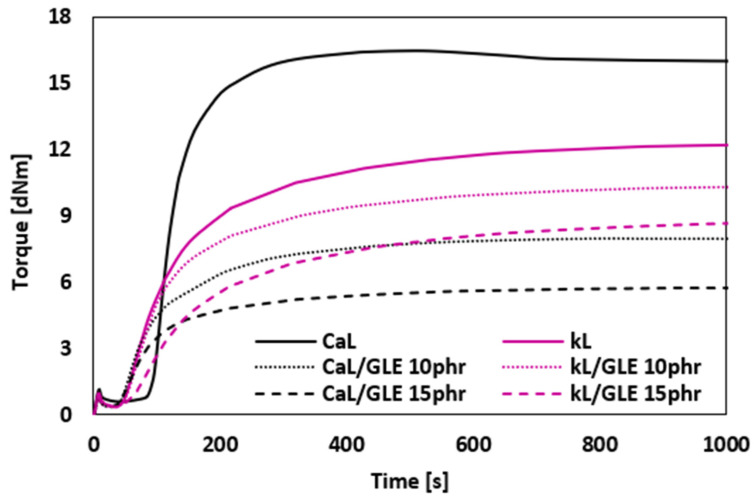
Vulcanization isotherms of rubber compounds plasticized with GLE.

**Figure 3:**
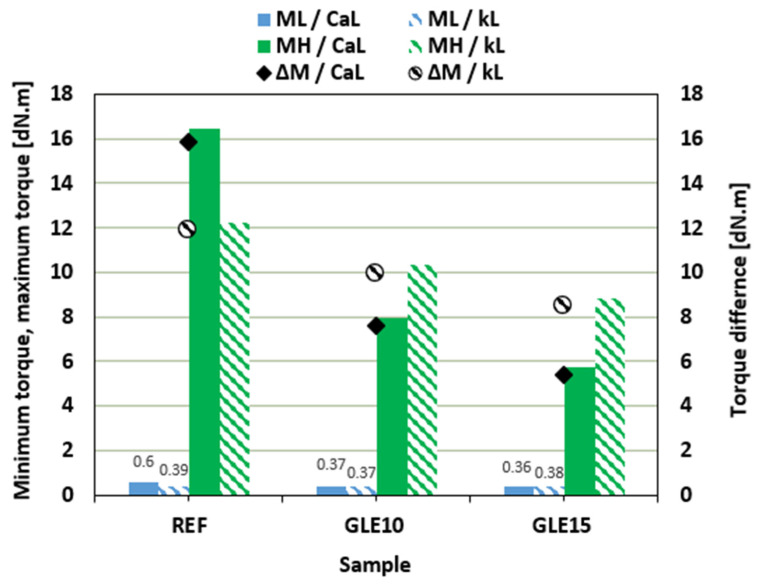
Influence of GLE on minimum *M_L_*, maximum *M_H_* torque and torque difference Δ*M* of rubber compounds.

The vulcanization isotherms of rubber compounds plasticized with GLE are presented in Figure 2, while the values of minimum *M_L_* and maximum *M_H_* torque, as well as torque difference Δ*M*, are summarized in Figure 3. It becomes apparent that the application of the plasticizer resulted in a decrease in the minimum, but mainly in the maximum torque. Subsequently, the torque difference decreased depending on the amount of the plasticizer. The reduction in minimum torque with GLE addition is linked to the lowered viscosity of the rubber compound prior to curing, whereas the reduction in maximum torque is associated with the decreased viscosity of the cured rubber compounds. It is evident that GLE functions as a plasticizer or softening agent for rubber composites. Its small molecules penetrate the spaces between molecules, interfering with the physical couplings both between and within macromolecules. This results in increased mobility of the rubber chains and decreased internal friction and viscosity of the compounds. From Figure 3, it is shown that higher *M_L_*, *M_H_* and Δ*M* were exhibited by the reference composite filled with CaL when compared to that filled with kL. Conversely, higher *M_H_* and Δ*M* of the compounds with applied GLE were demonstrated by those filled with kL. When compared to the reference samples, a much higher decrease in *M_H_* and Δ*M* in dependence on plasticizer content was observed for the composites filled with CaL. Only a moderate decrease in both characteristics was recorded for the composites filled with kL. This points to a much higher plasticizing effect of GLE on the rubber formulations filled with CaL.

**Figure 4:**
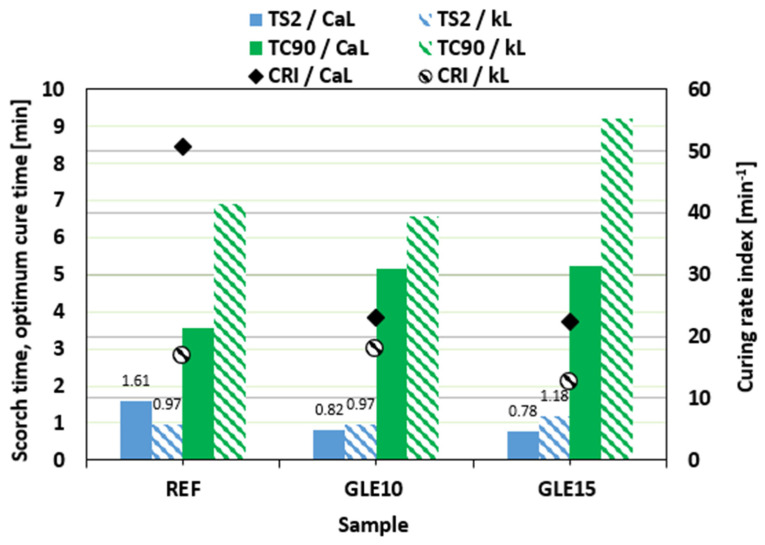
Influence of GLE on scorch time *t_s_*_2_, optimum cure time *t_c_*_90_, and curing rate index *CRI* of rubber compounds.

In comparison with the reference sample filled with CaL, the scorch time *t_s_*_2_ of the compounds with added GLE was slightly lower (Figure 4). Almost no change in scorch time was observed for rubber compounds filled with kL. On the other hand, as shown in Figure 4, the application of the plasticizer resulted in the prolongation of the optimum cure time *t_c_*_90_ for both CaL- and kL-filled formulations. Due to the extension of the optimum cure time, the curing rate index *CRI* decreased (Figure 4), markedly pointing to the deceleration of the vulcanization kinetics. Longer *t_c_*_90_ of the compounds filled with kL was reflected in a lower curing rate index of the equivalent composites.

**Figure 5:**
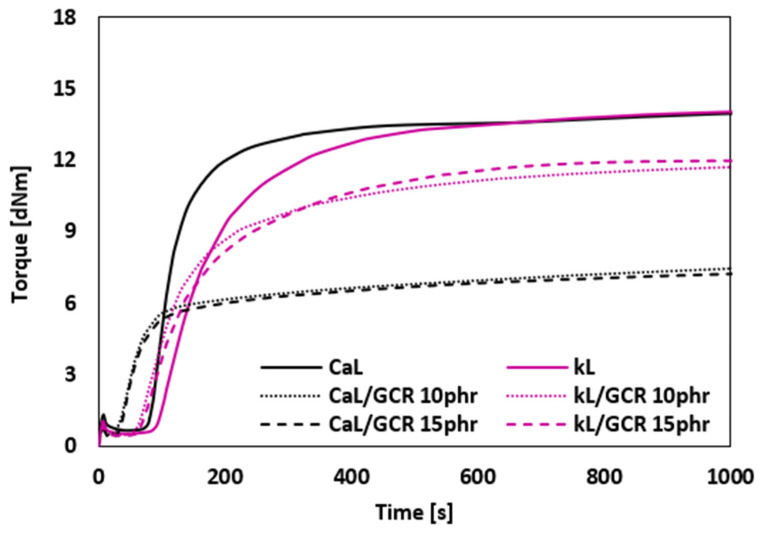
Vulcanization isotherms of rubber compounds plasticized with GCR.

**Figure 6:**
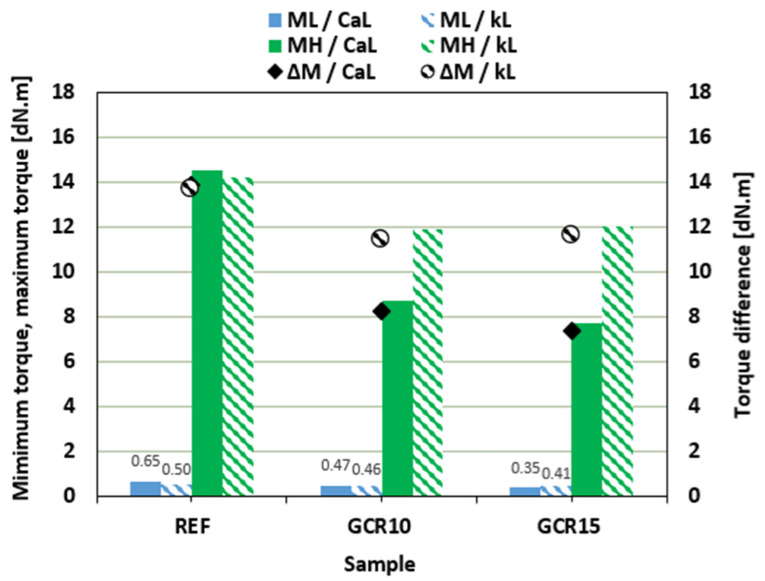
Influence of GCR on minimum *M_L_*, maximum *M_H_* torque and torque difference Δ*M* of rubber compounds.

Figure 5 depicts the vulcanization isotherms of the composite plasticized with GCR. It becomes apparent that the application of 10 phr plasticizer led to the decrease in the minimum and maximum torque as well as the torque difference. However, by the next increase in plasticizer content, no significant change in the course of the curing isotherms and torque difference was observed. As in the previous case, a higher decrease in maximum torque and torque difference in relation to the reference sample was recorded for composites based on CaL, again pointing to a higher plasticizing effect of GCR on CaL-filled rubber compounds. As shown in Figure 5, the course of the curing isotherms for both reference composites was very similar, unveiling similar values of *M_L_*, *M_H_* and Δ*M* (Figure 6). This points to a reduction in the differences in viscosities between both composites by an increase in the compounding temperature from 90 °C to 110 °C (see Section 3.2.3).

**Figure 7:**
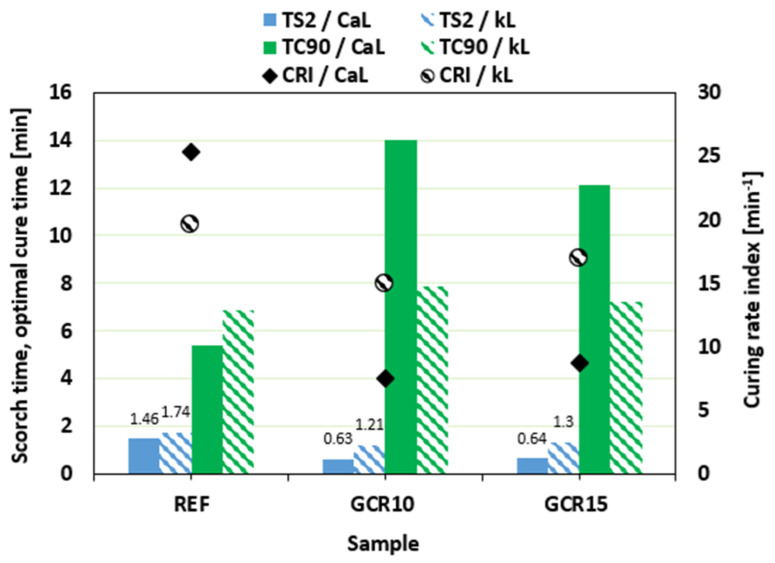
Influence of GCR on scorch time *t_s_*_2_, optimum cure time *t_c_*_90_, and curing rate index *CRI* of rubber compounds.

When compared to references, the scorch time was shortened by the addition of the plasticizer, which was more significant for rubber compounds filled with CaL. As shown in Figure 7, the *t_s_*_2_ of GCR plasticized compounds was reduced by more than half when compared to that of the reference. The influence of the plasticizer on the scorch time of composites filled with kL was minimal. Conversely, the plasticized rubber compounds required a longer time for their optimal cross-linking. The reference composite based on CaL exhibited shorter optimum cure time when compared to the equivalent kL-filled sample. On the other hand, the application of GCR caused much higher prolongation of *t_c_*_90_ for CaL-filled rubber compounds, pointing to their lower curing kinetics and lower curing rate index *CRI* (Figure 7).

The observed variations in scorch time and optimum cure time are primarily attributed to the plasticizing effect and the dilution of the rubber matrix by the oligomeric chains of GLE and GCR. Given the trace amounts of residual catalysts in the recycled products (below 10 ppm), any potential accelerating or retarding effect of Na^+^ or K^+^ ions on the sulfur vulcanization system can be considered statistically insignificant in the context of this study.

**Figure 8:**
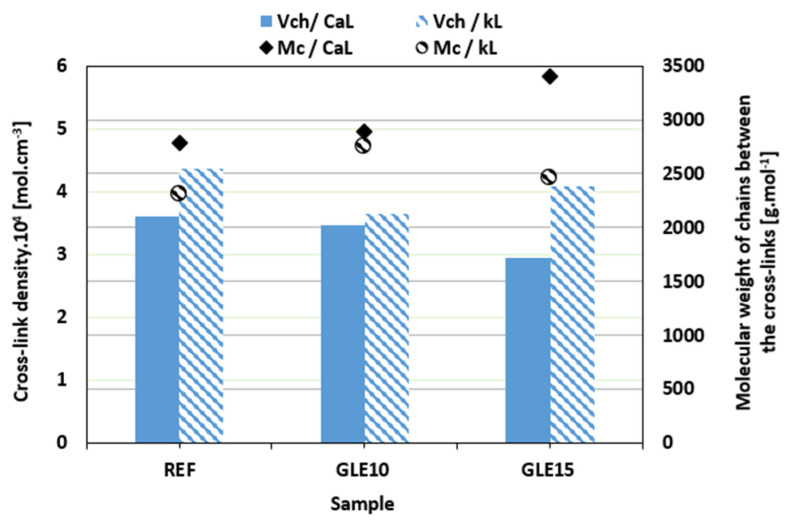
Influence of GLE on cross-link density *ν_ch_* and molecular weight of chains between the cross-links *M_c_* of composites.

**Figure 9:**
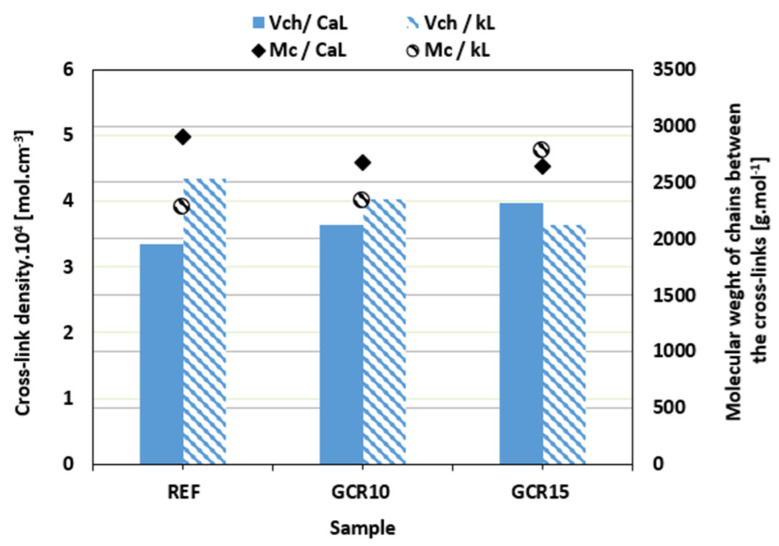
Influence of GCR on cross-link density *ν_ch_* and molecular weight of chains between the cross-links *M_c_* of composites.

The prolongation in the optimum cure time and the decrease in the curing rate index of the compounds with both tested plasticizers suggest that the application of plasticizers resulted in deceleration of the vulcanization process. The reason can be attributed to the fact that, due to the polar nature of GLE and GCR, both plasticizers can dilute or absorb curing agents, rendering them less effective during the curing process [40,53,54]. The deceleration of the curing process might influence the composites’ degree of cross-linking. Thus, the cross-link density *ν_ch_* was evaluated, and the results are summarized in Figure 8 and Figure 9. Looking at them, it can be deduced that the influence of plasticizers on the cross-link density of both CaL- and kL-filled composites was low. In some cases, the cross-link density slightly decreased or increased with no direct relation to the type of filler or plasticizer applied. It can also be stated that although the plasticizers caused the deceleration of the vulcanization process and, in most cases, a slight decrease in cross-link density, no significant effect on cross-linking degree was observed. With the exception of the composites plasticized with 15 phr of GCR, higher cross-link density was demonstrated in the composites filled with kL. The results of our previous experimental outputs showed that the tested biopolymer fillers do not participate in the formation of cross-links with the rubber matrices [38,55]. Instead, they can act as steric hindrances against the formation of the linkages between the adjacent chain segments. With a much higher molecular weight of CaL when compared to kL, lignosulfonate can be a bigger barrier, suppressing the creation of the cross-links. Having lower cross-link density, composites based on CaL demonstrated higher molecular weight of the chain segments between the cross-links *M_c_* (Figure 8 and Figure 9). The complete set of curing characteristics and cross-link density values for all investigated NBR compounds is summarized in Table 6 for a comprehensive overview and direct comparison.

#### 3.2.3. Rheology

**Figure 10:**
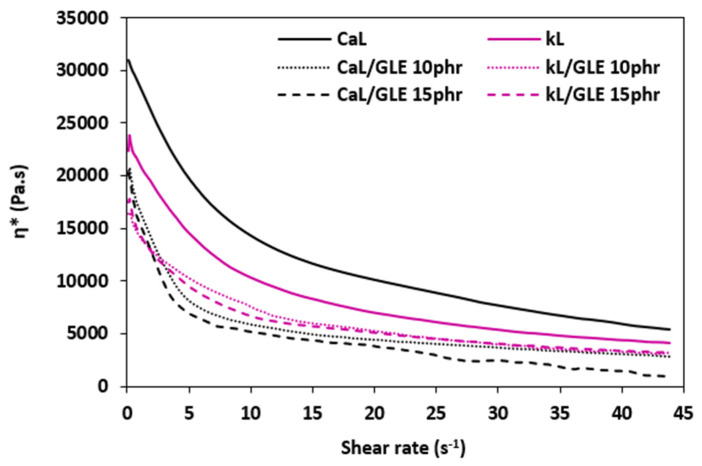
The dependence of dynamic complex viscosity η* on shear rate of composites plasticized with GLE.

**Figure 11:**
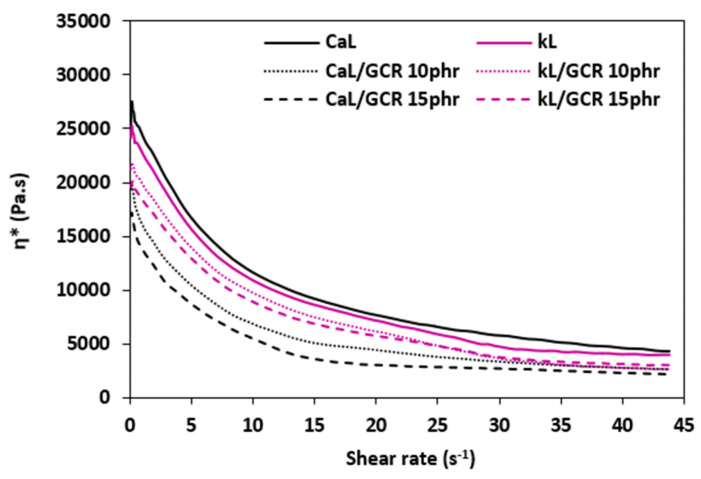
The dependence of dynamic complex viscosity η* on shear rate of composites plasticized with GCR.

The dependences of dynamic complex viscosities η* on applied shear rate for both composite types are presented in Figure 10 and Figure 11. The results obtained from rheological measurements confirmed a good correlation with the torque data summarized in the section above. As shown, the highest dynamic complex viscosities across the entire shear rate range were demonstrated by the reference compounds based on CaL, followed by the equivalent composites filled with kL. The higher viscosity of CaL-filled reference composites can be attributed to the higher molecular weight of lignosulfonate and likely its poorer dispersion within the composites. As higher differences in *M_H_* and Δ*M* were observed between reference composites compounded at 90 °C, higher differences in the dynamic complex viscosity of the equivalent composites were also recorded. Lower differences in *M_H_*, Δ*M*, and η* between reference composites compounded at 110 °C might be attributed to better dispersion and homogenization of the composite formulations due to their lower viscosity evoked by the higher mixing temperature. The addition of GLE and GCR resulted in a decrease in dynamic complex viscosities, which clearly confirmed their plasticizing effect on the rubber compounds. The higher the amount of plasticizers, the lower the viscosities. As also shown in Figure 10 and Figure 11, the differences in viscosities between reference samples and plasticized composites are more pronounced at lower shear rates. With an increase in shear rates, the differences in viscosities became less visible. In both composite types, plasticized rubber compounds filled with CaL exhibited lower viscosities when compared to those filled with kL, pointing to a much higher plasticizing effect of both plasticizers on CaL. The lowest viscosities were demonstrated by the CaL-filled composites plasticized with 15 phr of GLE or GCR. A possible explanation for the different plasticizing effects on the tested biopolymer fillers might be the different polarity and surface structure of CaL and kL. kL is typically regarded as hydrophobic (non-polar), whereas CaL is amphiphilic, possessing both polar and non-polar functionalities due to the presence of sulfonate groups. Highly polar, negatively charged sulfonate groups make CaL water-soluble, contributing to its much higher polarity and imparting it with surfactant properties. The tested plasticizers obtained from the chemical recycling of polyurethanes contain polar functional moieties, which rank them among polar materials. It is well known that polar plasticizers are more efficient for the plasticization of polar-based formulations. Consequently, it can be stated that the plasticizing effect of polar plasticizers (GLE, GCR) on rubber formulations based on polar NBR filled with highly polar CaL is much higher when compared to those filled with kL.

#### 3.2.4. Mechanical Properties

**Figure 12:**
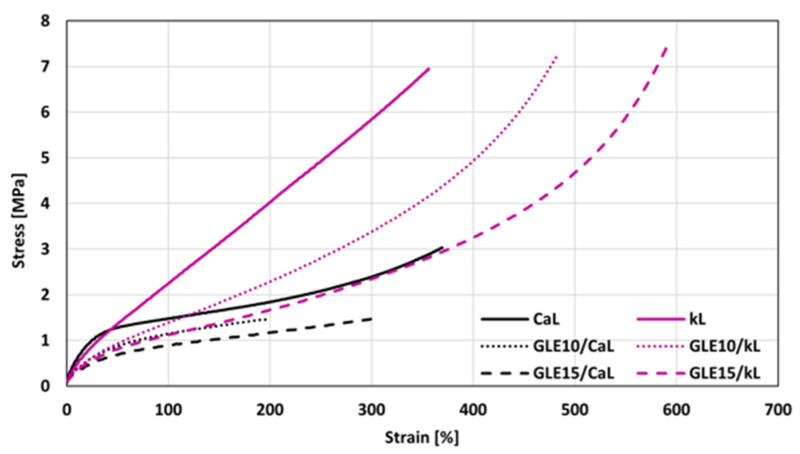
Stress–strain curves of composites plasticized with GLE.

**Figure 13:**
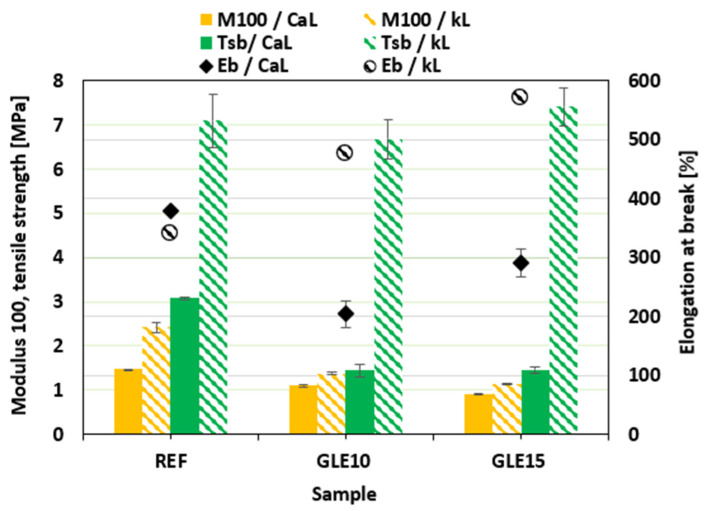
Influence of GLE on modulus M100, tensile strength Tsb and elongation at break Eb of composites.

The stress–strain curves of the composites plasticized with GLE are depicted in Figure 12, while the ultimate values of their mechanical properties are summarized in Figure 13. For a more precise comparison, the numerical values of tensile strength Tsb and elongation at break Eb for all investigated NBR formulations are also presented in Table 7. It becomes apparent that the modulus M100 of both CaL- and kL-filled composites showed a decreasing trend with increasing GLE content. A notable observation is the measurable decrease in modulus and torque difference despite the relatively stable chemical cross-link density. In rubber science, these parameters characterize different structural aspects of the composite. While *ν_ch_* (determined by equilibrium swelling) primarily reflects the concentration of stable chemical sulfur cross-links, the modulus and torque are influenced by a broader range of physical factors. This decrease in stiffness, while the chemical network remains intact, is attributed to the physical plasticizing effect of the recycled polyols. By increasing the free volume and enhancing the mobility of the macromolecular chain segments, GLE and GCR reduce the internal friction and resistance to deformation.

**Figure 14:**
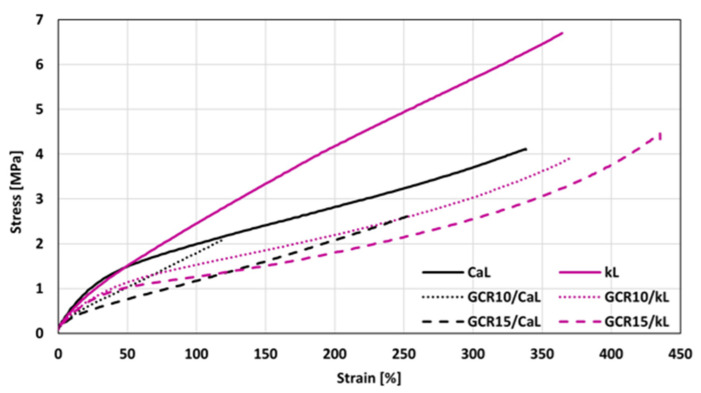
Stress–strain curves of composites plasticized with GCR.

**Figure 15:**
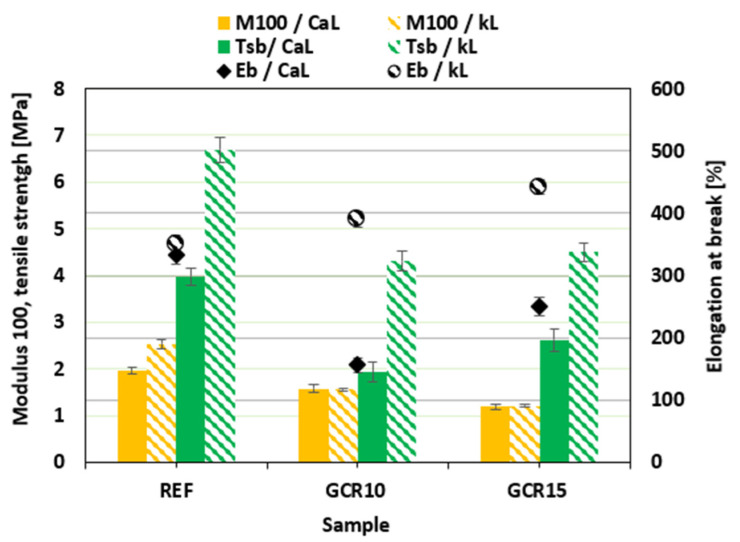
Influence of GCR on modulus M100, tensile strength Tsb and elongation at break Eb of composites.

Furthermore, the hydrodynamic effect and the nature of filler–rubber interactions play a crucial role. The incorporation of these oligomeric plasticizers improves the dispersion of lignin (as confirmed by SEM) and lubricates the filler–rubber interface. This reduction in filler–filler networking and improved interfacial lubrication leads to a lower overall stiffness of the vulcanizates. Thus, the stability of the cross-link density confirms that the recycled plasticizers are chemically compatible with the curing system and do not interfere with the sulfur vulcanization chemistry, while the changes in mechanical response are a direct consequence of their physical plasticizing and compatibilizing action. The higher modulus of the composites filled with kL can be attributed to their higher degree of cross-linking (Figure 8). Looking at Figure 12, one can see different stress–strain behavior for both CaL- and kL-filled composites at higher deformation forces. Both reference composites exhibited similar elongation at break, although the kL-filled sample demonstrated a more than twofold higher tensile strength (7.1 MPa for the kL reference vs. 3.1 MPa for the CaL reference). The application of 10 phr GLE led to a decrease in tensile strength as well as elongation at break for the CaL-filled composites. However, by increasing the plasticizer content to 15 phr, the tensile strength was maintained at a similar level, while an increase in elongation at break was observed. On the other hand, as shown in Figure 12 and Figure 13, the elongation at break of the kL-filled composites exhibited an increasing trend with the amount of GLE, rising from 341% for the reference to 571% for the composite plasticized with 15 phr. Moreover, no negative influence of the plasticizer on tensile strength was recorded, as the values fluctuated only within a narrow range close to the reference. Figure 14 depicts the stress–strain behavior of the composites plasticized with GCR, with the determined values of modulus M100, tensile strength, and elongation at break presented in Figure 15. Similar dependencies of mechanical properties on the type of filler were observed. The highest tensile strength was exhibited by the reference composite filled with kL, which was comparable to the corresponding reference sample compounded at 90 °C. Similarly, the modulus and elongation at break of the reference composite compounded at 110 °C were close to those of the equivalent reference formulated at 90 °C, indicating that the compounding temperature has no obvious influence on the properties of the kL-filled references. The addition of GCR caused a decrease in modulus and tensile strength, while increasing elongation at break. The GCR-plasticized composites filled with kL demonstrated lower tensile strength and elongation at break, but a similar modulus M100 compared to equivalent composites plasticized with GLE, suggesting that GLE is a more effective plasticizer for kL-filled rubber compounds. The influence of GCR on the tensile behavior of CaL-filled composites was very similar to that of the GLE-plasticized equivalents. As shown in Figure 15, the modulus decreased, while the tensile strength and elongation at break decreased at 10 phr loading before subsequently increasing at 15 phr. This non-linear behavior observed in the CaL-filled composites can be attributed to the competition between matrix dilution and filler dispersion efficiency. At 10 phr loading, the plasticizer likely causes a dilution effect in the rubber matrix, increasing free volume and decreasing strength, but is insufficient to fully plasticize the rigid, high-molecular-weight CaL aggregates. This state may represent a transitional phase where the plasticizer disrupts the existing filler–filler network without yet providing sufficient interfacial lubrication, leading to localized stress concentrations and the observed initial drop in both tensile strength and elongation at break. However, upon reaching 15 phr, a critical concentration is achieved where the plasticizer effectively penetrates the CaL structure, facilitating the breakdown of larger agglomerates into smaller domains and promoting a more flexible filler–rubber interface. This improved dispersion and enhanced interfacial compatibility, as supported by SEM observations, lead to the stabilization of tensile strength and an increase in elongation at break. GCR-plasticized composites filled with CaL manifested higher modulus and tensile strength, while having lower elongation at break in comparison with the corresponding CaL-filled composites plasticized with GLE.

The tensile strength values achieved for kL-filled composites plasticized with 15 phr of GLE (7.41 MPa) and GCR (4.50 MPa) are competitive within the context of technical NBR rubber applications. For NBR formulations containing conventional plasticizers (such as naphthenic oils or phthalate esters like DOP) at similar loading levels, typical tensile strength values range between 6 and 10 MPa, with elongation at break typically between 300% and 600%. Our results, particularly for the kL/GLE_15_ system (Tsb = 7.41 MPa, Eb = 571%), fall well within these industrial benchmarks. The high elongation at break suggests that GLE and GCR provide an effective plasticizing action comparable to petrochemical alternatives, while offering the added benefit of being derived from recycled polyurethane waste.

Beyond basic mechanical properties, the chemical structure of GLE and GCR suggests additional functional advantages over conventional mineral oils. Due to their higher polarity and oligomeric nature (average molecular weight), these recycled plasticizers are expected to exhibit lower migration rates from the NBR matrix—a common issue with low-molecular-weight paraffinic or naphthenic oils. Furthermore, the presence of residual urethane and hydroxyl groups may contribute to improved thermal aging resistance and oil resistance, similar to the behavior of specialized polyester plasticizers. This is further supported by the thermal stability data (TGA), where the 5% weight loss temperature (T_5%_) for GLE (330 °C) and GCR (255 °C) remains within or above the typical range for conventional rubber process oils (200–340 °C), ensuring their viability for standard vulcanization and service conditions.

In a broader context, the performance of the investigated GLE and GCR plasticizers highlights significant advantages over conventional petroleum-based additives. Compared to standard mineral oils or TDAE, these recycled polyols exhibit superior thermal stability (as shown in Table 4), ensuring a wider processing window and lower volatility during compounding. Furthermore, while conventional plasticizers often lead to a significant drop in tensile strength due to simple matrix dilution, the polar nature of GLE and GCR (due to hydroxyl and urethane groups) allows them to act as interfacial modifiers. This is particularly evident in the kL-filled systems, where GLE effectively balanced flexibility and strength, matching or exceeding results reported for conventional NBR formulations in the recent literature [40,56,57]. This dual functionality—acting as both a softening agent and a compatibility enhancer—positions these waste-derived polyols as a sustainable and technically viable alternative to commercial plasticizers in polar rubber matrices.

#### 3.2.5. Morphology

**Figure 16:**
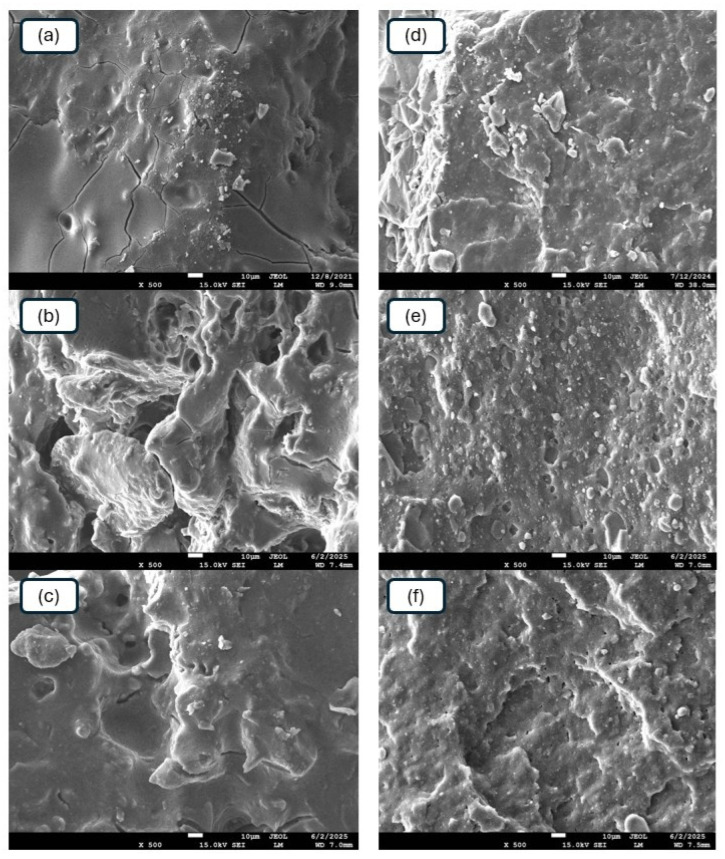
SEM images of reference composite filled with CaL (**a**), CaL-filled composite plasticized with 10 phr of GLE (**b**), CaL-filled composite plasticized with 15 phr of GLE (**c**), reference composite filled with kL (**d**), kL-filled composite plasticized with 10 phr of GLE (**e**), kL-filled composite plasticized with 15 phr of GLE (**f**).

**Figure 17:**
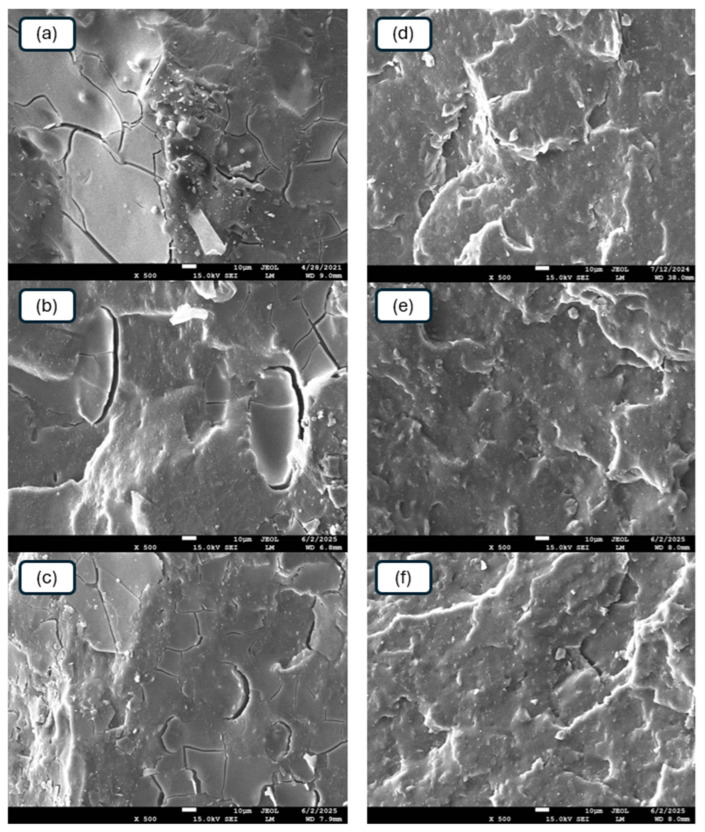
SEM images of reference composite filled with CaL (**a**), CaL-filled composite plasticized with 10 phr of GCR (**b**), CaL-filled composite plasticized with 15 phr of GCR (**c**), reference composite filled with kL (**d**), kL-filled composite plasticized with 10 phr of GCR (**e**), kL-filled composite plasticized with 15 phr of GCR (**f**).

Scanning electron microscopy was performed to study the morphology and microstructure of the composites. SEM images of the reference samples and composites plasticized with GLE are presented in Figure 16, while microscopic images of their equivalents with applied GCR are depicted in Figure 17.

From Figure 16, it becomes apparent that both CaL and kL tended to form agglomerates in the reference composites. A detailed examination of these micrographs (Figure 16a,d) revealed a coarse morphology with large lignin agglomerates, where some domains reached diameters of approximately 8–15 µm. Upon the application of 10 phr GLE, CaL still formed large domains (Figure 16b), while at 15 phr GLE, a better dispersion of the filler was observed (Figure 16c). Manual quantitative assessment of the micrographs indicates that with 15 phr of GLE, the average diameter of the visible filler clusters significantly decreased to below 3–5 µm. Simultaneously, better adhesion and compatibility between the rubber and the filler were achieved. On the other hand, GLE-plasticized composites filled with kL demonstrated a more homogeneous and compact structure (Figure 16e,f). Good dispersion and distribution of kL were observed within the composites. kL formed small, well-dispersed domains showing enhanced interfacial compatibility with the NBR matrix. This improved adhesion, evidenced by the more homogeneous morphology in the SEM micrographs and the significantly higher tensile strength compared to the CaL-filled systems, facilitates more effective stress transfer between the elastomer and the filler phase.

A similar situation was observed in the case of composite plasticized with GCR (Figure 17). The addition of the plasticizer led to better dispersion of both CaL and kL. Better homogeneity and adhesion between the rubber and the fillers at their interface were observed. In the reference samples (Figure 17a,d), large filler aggregates were again identified (up to 12 µm), whereas the incorporation of 15 phr GCR (Figure 17c,f) resulted in a much finer dispersion, with the majority of filler domains remaining in the sub-micron or low micron range (approx. 2–3 µm). Again, the GCR-plasticized composites filled with kL exhibited a more compact structure with better filler–rubber interfacial adhesion.

As outlined, GLE and GCR are low molecular weight substances derived from the chemical recycling of polyurethane, which plasticize both kL and CaL. This leads to their plasticization and a reduction in viscosity. Concurrently, the tested plasticizers exert a softening effect on the rubber matrix. Consequently, the viscosity of the rubber compounds decreased (Figure 10 and Figure 11). The viscosities of both the matrix and the filler became closer, enhancing the compatibility and miscibility between the two components during the mixing phase. This resulted in more effective dispersion and distribution of the biopolymers within the rubber compounds, as well as increased adhesion and homogeneity at the interface between the biopolymers and the rubber matrix. Both plasticizers are also proposed to serve as rubber–filler interfacial compatibilizers, contributing to the enhanced compatibility and adhesion between the two components. In general, higher mechanical characteristics were demonstrated by composites filled with kL. It was noted that the influence of plasticizers on the viscosity reduction in kL-filled compounds was less evident. However, kL exhibited a much lower molecular weight compared to CaL, which very likely contributed to its much better dispersion and distribution within the rubber matrix and the formation of a more robust and effectively integrated filler–rubber network. 

#### 3.2.6. Dynamic Mechanical Analysis

**Figure 18:**
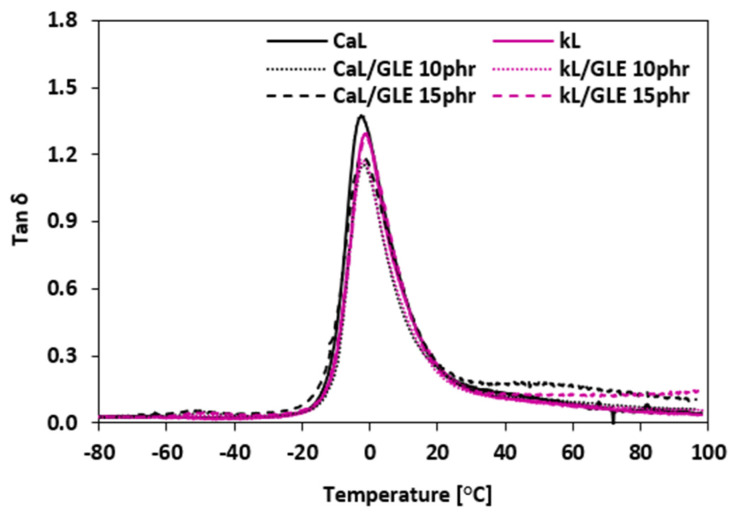
The temperature dependencies of the loss factor *tan δ* of composites plasticized with GLE.

**Figure 19:**
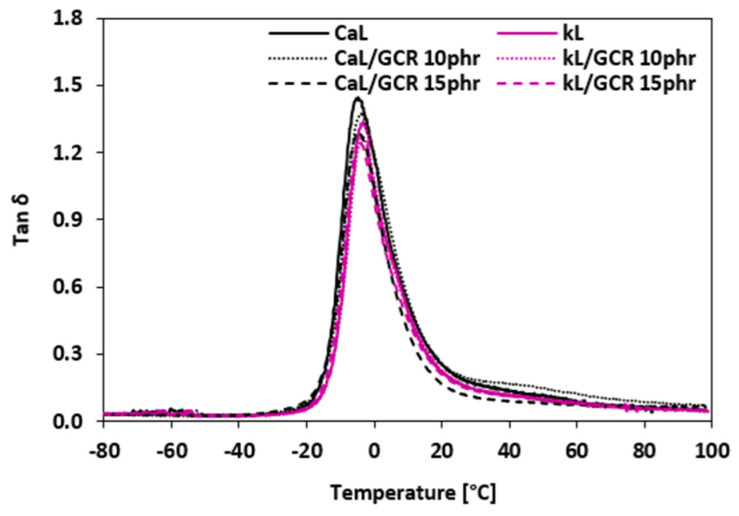
The temperature dependencies of the loss factor *tan δ* of composites plasticized with GCR.

Dynamic mechanical analysis (DMA) was conducted to assess the impact of the biopolymer fillers and recycled plasticizers on the viscoelastic characteristics of the NBR composites. The temperature dependencies of the loss factor (*tan δ*) for composites plasticized with GLE and GCR are shown in Figure 18 and Figure 19, respectively. The peak maxima in the *tan δ* curves correspond to the glass transition temperature (*Tg*). The *Tg* values, along with *tan δ* and storage modulus (*E*′) values at selected temperatures, are summarized in Table 8, Table 9, Table 10 and Table 11. As indicated in Table 7 and Table 9, the application of GLE and GCR had almost no influence on the *Tg* of the composites, regardless of the filler type (CaL or kL), as the values fluctuated only within a very narrow experimental range (from –4.6 °C to –1.0 °C). This suggests that the recycled polyols do not significantly interfere with the segmental mobility of the NBR matrix at the glass transition. In the glassy region (–50 °C to –20 °C), the *tan δ* values were consistently low and similar for all samples, indicating high stiffness and limited molecular motion. In the transition region, reference composites based on both biopolymer fillers exhibited the highest *tan δ* at *Tg*. The application of plasticizers resulted in a slight reduction in the loss factor peak height. Lower *tan δ* values in this region indicate that the transition from the glassy to the rubbery state is more efficient and less energy-demanding. This behavior is likely related to the improved dispersion of kL and CaL and enhanced interfacial adhesion (as seen in SEM, Figure 16 and Figure 17), which reduces internal friction and the presence of microcavities at the filler–rubber interface. A critical aspect of this analysis is the correlation between the dynamic storage modulus (*E*′) and the static tensile modulus (M100). The observed trends are consistent with the literature reports on rubber materials [58], where a clear relationship is typically demonstrated between the dynamic stiffness in the rubbery region and the static mechanical performance. As summarized in Table 8 and Table 10, a clear relationship is demonstrated between the dynamic stiffness in the rubbery region (20 °C and 50 °C) and the static mechanical data discussed in Section 3.2.4. Specifically, the decrease in M100 following the addition of recycled polyols is fundamentally validated by a corresponding decrease in *E*′. For instance, in kL-filled systems, the reduction in M100 from 2.42 MPa to 1.15 MPa (with 15 phr GLE) corresponds to a drop in *E*′ at 20 °C from 13.9 MPa to 8.0 MPa. This correlation confirms that the decrease in static stiffness is not an isolated artifact of tensile testing but a fundamental change in the material’s viscoelastic resistance to deformation. The significant reduction in complex viscosity (*η∗*) discussed in Section 3.2.3 provides a strong mechanistic basis for this decrease in *E*′. While E′ reflects the elastic energy storage capacity, its reduction in the rubbery region mirrors the lower internal resistance to flow and increased molecular mobility provided by the plasticizers. In the rubbery region (20 °C and 50 °C), a slight increase or stabilization in *tan δ* was observed with increasing plasticizer content, particularly for the CaL/GCR_15_ sample. This trend further confirms the increased molecular mobility of the NBR chains. Furthermore, the slightly lower *Tg* observed for composites compounded at 110 °C compared to those at 90 °C can be attributed to the higher molecular mobility of the chain segments achieved at higher mixing temperatures. The enhanced interfacial adhesion observed in kL systems (Figure 16 and Figure 17) and their higher tensile strength (Figure 13 and Figure 15) confirm efficient stress transfer. This improved reinforcement partially compensates for the softening effect of GLE and GCR, leading to the balanced viscoelastic performance of the final composites.

#### 3.2.7. Sustainability and Application Potential

The utilization of recycled GLE and GCR plasticizers represents a strategic approach to raw material recovery, effectively reducing the reliance on conventional petrochemical additives. Although a full life cycle assessment (LCA) was beyond the scope of this initial material characterization study, the environmental potential of such circular economy strategies is well-supported by the established literature. Scientific reports indicate that chemical recycling of polymer waste can significantly reduce environmental impact compared to virgin material production. For instance, chemical recycling of mixed plastics has been shown to achieve approximately 50% lower global warming potential (GWP) compared to alternative disposal processes, with recycled plastic production generating substantially fewer CO_2_ equivalent emissions than virgin polymers [59,60]. Furthermore, the incorporation of recovered recyclates into composite systems can reduce the climate impact by over 22% compared to systems based on virgin fibers [61]. In the context of the rubber industry, replacing petroleum-based process oils with GLE and GCR aligns with “green chemistry” principles by closing the loop in polyurethane waste management. By repurposing this waste into functional additives, the investigated NBR composites potentially contribute to a lower overall carbon footprint and reduced dependence on fossil resources. Future research will focus on a quantitative evaluation of these environmental benefits through a comprehensive LCA using specialized software, providing the necessary indicators to validate these sustainable formulations on an industrial scale.

## 4. Conclusions

Two types of recycled polyols, GLE and GCR, were successfully implemented as sustainable plasticizers for NBR-based compounds filled with kL and CaL. This study demonstrates a viable technological pathway for the development of rubber composites incorporating both biomass-derived fillers and chemically recycled polyurethane waste. Rheological analysis confirmed a robust plasticizing effect, evidenced by a significant downward shift in complex viscosity curves across the entire shear rate range investigated. While the plasticizers slightly prolonged the optimum cure time, they maintained a stable chemical cross-link density, ensuring the structural integrity of the rubber network and consistent processing behavior. The synergistic effect of recycled polyols was most evident in the morphology and mechanical performance of the composites. SEM analysis confirmed that the plasticizers acted as effective dispersing agents, facilitating the breakdown of biopolymer agglomerates and leading to more homogeneous dispersions with enhanced interfacial compatibility. The optimal performance was achieved by the combination of kL and GLE, which maintained a high tensile strength of approximately 7–8 MPa while significantly improving elongation at break compared to the unplasticized reference. In contrast, CaL-filled composites exhibited lower mechanical stability (approx. 3 MPa) due to the higher molecular weight and steric hindrance of the filler. Dynamic mechanical analysis showed that the plasticizers did not significantly shift the glass transition temperature, which remained stable between −2.5 °C and −1 °C for GLE-based systems and between −4.6 °C and −3.1 °C for GCR-based systems, ensuring that the low-temperature flexibility of the NBR matrix was preserved.

The polar nature of the recycled polyols provides a dual advantage: improved compatibility with the NBR matrix compared to traditional non-polar mineral oils and enhanced efficiency in dispersing polar biopolymeric fillers. While the utilization of waste resources aligns with circular economy principles, it is important to note that the overall environmental sustainability of these composites depends on various factors, including the energy consumption of the glycolysis process and the life cycle of the chemical agents used. Ultimately, these findings provide a foundational step toward reducing the reliance on fossil-based additives in the rubber industry by validating the functional potential of chemically recycled polyurethane waste in high-performance NBR composites.

## Figures and Tables

**Table 1:** Characteristics of CaL and kL.

Characteristic	CaL (Borrement CA120)	kL (BioPiva 100)
Molecular weight (Mw)	~24,000 g·mol^−1^	~5000 g·mol^−1^
Phenolic hydroxyl groups	1.56%	2.9%
Aliphatic hydroxyl groups	-	1.02%
Methoxy groups	-	2.4%
Carboxy groups	-	0.31%
Calcium content	5%	-
Sulfur content	7%	-
Ash content	-	<2%
Specific surface area	3.9 m^2^/g	-

**Table 2:** Quantitative molecular weight distribution of the GCR recyclate determined by GPC.

Peak	Retention Time (Min)	Mw (g·mol^−1^)
1	13.1	1721
2	14.2	777
3	14.8	518
4	15.9	385
5	16.8	282
6	17.4	108

**Table 3:** Rubber compound composition expressed in phr and compounding temperatures.

Sample Designation	NBR	CaL	kL	GLE	GCR	Mixing Temperature
CaL ref.	100	30				90/110 °C
kL ref.			30			90/110 °C
CaL/GLE_10_		30		10		90 °C
CaL/GLE_15_		30		15		90 °C
kL/GLE_10_			30	10		90 °C
kL/GLE_15_			30	15		90 °C
CaL/GCR_10_		30			10	110 °C
CaL/GCR_15_		30			15	110 °C
kL/GCR_10_			30		10	110 °C
kL/GCR_15_			30		15	110 °C

phr = parts per hundred parts of rubber (by weight). All compounds contain a constant curing system: 3 phr sulfur, 1.5 phr CBS (accelerator), 3 phr ZnO, and 2 phr stearic acid (activators).

**Table 4:** The characteristics of plasticizers GLE and GCR.

	Density [g·cm^−3^]	Viscosity @ 45 °C [Pa·s]	Viscosity @ 50 °C [Pa·s]	Hydroxyl Number [mg KOH·g^−1^]
GLE	1.040	10.4	3.00	148
GCR	1.174	3.46	2.96	114

**Table 5:** Thermal degradation parameters of recycled GLE and GCR plasticizers.

Sample	T_5%_ [°C]	T_10%_ [°C]	T_50%_ [°C]	Residual Mass at 800 °C [%]
GLE	338	355	390	1.8
GCR	255	290	365	4.2

**Table 6:** Curing characteristics and cross-link density of composites.

Sample	*M_L_* [dN·m]	*M_H_* [dN·m]	Δ*M* [dN·m]	*t_s_*_2_ [min]	*t_c_*_90_ [min]	*CRI* [min^−1^]	*ν_ch_*·10^4^ [mol·cm^−3^]	*M_c_* [g·mol^−1^]
CaL ref. (90 °C)	0.6	16.45	15.85	1.61	3.58	50.76	3.609	2782
kL ref. (90 °C)	0.39	12.25	11.86	0.97	6.9	16.86	4.367	2302
CaL/GLE_10_	0.37	7.97	7.6	0.82	5.17	22.99	3.465	2887
CaL/GLE_15_	0.36	5.74	5.38	0.78	5.25	22.37	2.945	3401
kL/GLE_10_	0.37	10.31	9.94	0.97	6.58	17.83	3.643	2745
kL/GLE_15_	0.38	8.83	8.45	1.18	9.21	12.45	4.078	2455
CaL ref. (110 °C)	0.65	14.49	13.84	1.46	5.40	25.38	3.353	2905
kL ref. (110 °C)	0.50	14.19	13.69	1.74	6.86	19.53	4.352	2272
CaL/GCR_10_	0.47	8.7	8.23	0.63	14.00	7.48	3.632	2677
CaL/GCR_15_	0.35	7.7	7.35	0.64	12.13	8.70	3.967	2640
kL/GCR_10_	0.46	11.87	11.41	1.21	7.89	14.97	4.034	2329
kL/GCR_15_	0.41	11.99	11.58	1.3	7.21	16.92	3.637	2776

**Table 7:** Mechanical properties of investigated composites.

Sample	Tsb [MPa]	Eb [%]
CaL ref. (90 °C)	3.09 ± 0.03	379.57 ± 3.38
kL ref. (90 °C)	7.10 ± 0.59	340.89 ± 34.06
CaL/GLE_10_	1.44 ± 0.14	203.74 ± 22.25
CaL/GLE_15_	1.46 ± 0.07	291.58 ± 23.92
kL/GLE_10_	6.68 ± 0.44	475.71 ± 17.77
kL/GLE_15_	7.41 ± 0.44	571.27 ± 13.86
CaL ref. (110 °C)	3.98 ± 0.19	332.97 ± 14.15
kL ref. (110 °C)	6.70 ± 0.27	350.94 ± 14.43
CaL/GCR_10_	1.94 ± 0.21	156.14 ± 11.51
CaL/GCR_15_	2.62 ± 0.25	249.90 ± 15.53
kL/GCR_10_	4.31 ± 0.21	390.58 ± 13.16
kL/GCR_15_	4.50 ± 0.20	441.59 ± 11.47

**Table 8:** Glass transition temperature *Tg* and loss factor *tan δ* of composites plasticized with GLE.

Sample	*Tg* (°C)	*tan δ* at *Tg*	*tan δ* (−50 °C)	*tan δ* (−20 °C)	*tan δ* (0 °C)	*tan δ* (20 °C)	*tan δ* (50 °C)
CaL	−2.5	1.37	0.03	0.05	1.27	0.25	0.11
CaL/GLE 10 phr	−1.6	1.15	0.05	0.05	1.10	0.24	0.11
CaL/GLE 15 phr	−2.1	1.19	0.05	0.08	1.13	0.27	0.17
kL	−1.1	1.29	0.02	0.05	1.26	0.26	0.10
kL/GLE 10 phr	−1.5	1.17	0.03	0.05	1.12	0.23	0.10
kL/GLE 15 phr	−1.0	1.28	0.05	0.06	1.25	0.24	0.13

**Table 9:** Storage modulus *E*′ (MPa) and modulus M100 (MPa) of composites plasticized with GLE.

Sample	*E*′ at *Tg*	*E*′ (−50 °C)	*E*′ (−20 °C)	*E*′ (0 °C)	*E*′ (20 °C)	*E*′ (50 °C)	*M*100
CaL	103	2984	2475	52.1	7.5	6.5	1.47
CaL/GLE 10 phr	107	2713	2044	68.5	10.6	10.0	1.10
CaL/GLE 15 phr	68	2136	1511	38.6	5.4	3.5	0.91
kL	130	3344	2829	96.4	13.9	12.6	2.42
kL/GLE 10 phr	144	3189	2509	94.9	15.5	14.9	1.38
kL/GLE 15 phr	94	3057	2184	67.7	8.0	4.7	1.15

**Table 10:** Glass transition temperature *Tg* and loss factor *tan δ* of composites plasticized with GCR.

Sample	*Tg* (°C)	*tan δ* at *Tg*	*tan δ* (−50 °C)	*tan δ* (−20 °C)	*tan δ* (0 °C)	*tan δ* (20 °C)	*tan δ* (50 °C)
CaL	−4.6	1.44	0.02	0.07	1.18	0.25	0.11
CaL/GCR 10 phr	−3.6	1.38	0.03	0.08	1.18	0.26	0.15
CaL/GCR 15 phr	−4.6	1.29	0.02	0.10	1.15	0.27	0.18
kL	−3.1	1.33	0.02	0.05	1.04	0.22	0.09
kL/GCR 10 phr	−3.2	1.28	0.02	0.06	1.08	0.23	0.10
kL/GCR 15 phr	−4.6	1.25	0.02	0.06	0.98	0.21	0.09

**Table 11:** Storage modulus *E*′ (MPa) and modulus M100 (MPa) of composites plasticized with GCR.

Sample	*E*′ at *Tg*	*E*′ (−50 °C)	*E*′ (−20 °C)	*E*′ (0 °C)	*E*′ (20 °C)	*E*′ (50 °C)	*M*100
CaL	87	3263	2650	33.3	7.5	6.5	1.96
CaL/GCR 10 phr	89	2952	2173	36.1	7.3	5.7	1.58
CaL/GCR 15 phr	95	2673	1932	35.5	7.6	5.9	1.19
kL	125	3253	2747	43.7	12.1	12.5	2.54
kL/GCR 10 phr	115	2824	2293	53.0	10.7	10.0	1.56
kL/GCR 15 phr	140	2958	2381	46.6	11.6	11	1.21

## Data Availability

The original contributions presented in this study are included in the article. Further inquiries can be directed to the corresponding authors.
